# Clinical prediction models to support the diagnosis of asthma in primary care: a systematic review protocol

**DOI:** 10.1038/s41533-018-0086-6

**Published:** 2018-05-18

**Authors:** L. Daines, S. McLean, A. Buelo, S. Lewis, A. Sheikh, H. Pinnock

**Affiliations:** 1 Asthma UK Centre for Applied Research, Usher Institute of Population Health Sciences and Informatics, The University of Edinburgh, Edinburgh, Scotland; 2 Scottish Collaboration for Public Health Research and Policy, The University of Edinburgh, Edinburgh, Scotland; 3 Edinburgh Clinical Trials Unit, Usher Institute of Population Health Sciences and Informatics, The University of Edinburgh, Edinburgh, Scotland

## Abstract

Substantial over-diagnosis and under-diagnosis of asthma in adults and children has recently been reported. As asthma is mostly diagnosed in non-specialist settings, a clinical prediction model (CPM) to aid the diagnosis of asthma in primary care may help improve diagnostic accuracy. We aim to systematically identify, describe, compare, and synthesise existing CPMs designed to support the diagnosis of asthma in children and adults presenting with symptoms suggestive of the disease, in primary care settings or equivalent populations. We will systematically search Medline, Embase and CINAHL from 1 January 1990 to present. Any CPM derived for use in a primary care population will be included. Equivalent populations in countries without a developed primary care service will also be included. The probability of asthma diagnosis will be the primary outcome. We will include CPMs designed for use in clinical practice to aid the diagnostic decision making of a healthcare professional during the assessment of an individual with symptoms suggestive of asthma. We will include derivation studies, and external model validation studies. Two reviewers will independently screen titles/abstracts and full texts for eligibility and extract data from included papers. The CHARMS checklist (or PROBAST if available) will be used to assess risk of bias within each study. Results will be summarised by narrative synthesis with meta-analyses completed if possible. This systematic review will provide comprehensive information about existing CPMs for the diagnosis of asthma in primary care and will inform the development of a future diagnostic model.

## Background

Asthma is a chronic respiratory disease affecting an estimated 334 million people worldwide.^1^ Progress towards improving asthma outcomes has been limited over the past decade.^2^ A key determinant in providing optimal asthma care is accurate and timely diagnosis. Yet, asthma remains a challenging disease to diagnose and substantial over-diagnosis and under-diagnosis of asthma in adults and children is reported.^3–5^ The consequences of under-diagnosis are lack of treatment, avoidable morbidity and mortality; while over-diagnosis leads to costly, potentially harmful treatment and unnecessary healthcare use. Furthermore, to realise the potential of precision medicine, greater accuracy in determining asthma traits at the point of diagnosis is required.^2^

Accurate diagnosis is challenging in a condition defined by its variability, in which the diagnosis is clinical, and in which symptoms, signs, and tests have poor predictive value for the diagnosis.^6–8^ The heterogeneity of asthma and recognition of distinct phenotypes and endotypes, adds further complexity,^2,9^ particularly as variable airflow obstruction remains the core diagnostic feature of asthma in current guidelines.^7,8^

Clinical prediction models (CPM), also referred to as prediction rules or risk scores^10^ combine at least two predictors, such as elements from a clinical history, physical examination, test results or response to treatment, to estimate the probability that a certain outcome is present.^10,11^ In clinical practice, CPMs can assist healthcare professionals to weigh up the probability of a diagnosis, enhance decision making with patients, or aid patient stratification.^12–14^

The current diagnostic strategies recommended in asthma guidelines are based on expert consensus and in some instances informed by health economic modelling, yet the accuracy with which the advocated clinical assessment pathways result in a correct diagnosis have not been formally evaluated.^7,8,15^

A prospectively validated CPM to support the diagnosis of asthma would offer an evidence-based approach for the assessment of individuals with symptoms suggestive of asthma. As most asthma diagnoses occur in non-specialist settings,^2^ a CPM developed for use in primary care (or equivalent settings where undifferentiated health problems are presented to healthcare professionals)^16^ would be particularly valuable.

To our knowledge, there are few CPMs to support the diagnosis of asthma in primary care. Previous searches identified a symptom-based diagnostic tool from South Korea,^17^ and an algorithm derived from Japanese healthcare;^18^ neither have been externally validated. A decision tree used to differentiate obstructive respiratory conditions has been derived and validated in the Netherlands.^19^ To build on these foundations, inform future development and progress towards wider implementation of a CPM for asthma diagnosis in primary care, a systematic review is needed.

## Aims

To identify, describe, compare, and synthesise all CPMs designed to support the diagnosis of asthma in children and adults presenting with symptoms suggestive of the disease, in primary care settings or equivalent populations.

## Discussion

Accurately diagnosing asthma is pivotal for effective symptom control and prevention of asthma attacks. By conducting this systematic review of CPMs for the diagnosis of asthma in primary care, we will critically appraise each included model, understand the strengths and limitations of each study design and the utility of each candidate predictor used, including signs and symptoms, personal and family history, and results from past and current clinical tests.

Although a CPM offers potential for improving the diagnosis of asthma, a number of questions need to be addressed before it will be accepted into clinical practice. Of fundamental importance is the outcome measure used to derive the model. In the absence of a “golden” reference standard for asthma diagnosis, the outcome measure must be carefully considered if an accurate diagnosis is to be made. Second, regardless of the statistical accuracy of a model, to be clinically useful, its impact on clinical outcomes should be tested, and healthcare professionals must trust the model and want to use it.^20,21^ Finally, contemporary understanding of asthma as an aggregate diagnosis^2,22^ suggests that a future diagnostic CPM may align with a precision medicine approach, potentially being able to stratify individuals by treatable traits.^9^ Our systematic approach will provide comprehensive information about existing CPMs, and inform the future development of a model for the diagnosis of asthma in primary care.

## Methods

The CHecklist for critical Appraisal and data extraction for systematic Reviews of prediction Modelling Studies (CHARMS) has been used to define the review question, and develop the review design.^23^ The Preferred Reporting Items for Systematic Review and Meta-Analysis Protocols guided protocol reporting.^24^

### Data sources and search strategy

We will complete a systematic search to identify all studies describing CPMs to support the diagnosis of asthma in primary care settings. In this review, a CPM is defined as any tool that combines at least two predictors, to provide an estimate for the probability of an outcome.^10,14^

Medline, Embase, and CINAHL databases will be searched from 1 January 1990 to present. The search strategy (Appendix 1) uses Cochrane asthma search syntax, with established search filters for prediction models.^25,26^ CPMs published in national clinical guidelines will be sought in TRIP (https://www.tripdatabase.com) and Guidelines Clearinghouse (https://www.guideline.gov). Forward and backward citations from the included studies will be searched for additional eligible studies. International experts and authors known to have published relevant work will be contacted to identify further studies. No language restrictions will be used. All studies will be translated into English where possible, any literature that we are unable to translate will be declared.

### Study eligibility criteria

#### Types of study

We will include derivation studies with or without external validation, in addition to external model validation studies with or without model updating.^23^

#### Population

Any CPM derived for use in a primary care population (or equivalent) will be included.

#### Intervention (type of model)

We will include CPMs designed for use in clinical practice to aid the diagnostic decision making of a healthcare professional during the assessment of an individual with symptoms suggestive of asthma.

#### Comparator

There are no relevant comparators for this systematic review.

#### Outcome to be predicted

The primary outcome is the probability of asthma diagnosis. Included studies should present at least one prediction model, or equivalent statistical method, in such a way that allows the probability of asthma to be calculated for other individuals. Some CPMs may use the broader outcome of obstructive airways disease. These studies will be included if data pertaining to asthma diagnosis can be extracted. CPMs derived for the diagnosis of occupational asthma will be included if the intended use is to aid diagnostic decision making in patients with undifferentiated symptoms.

#### Reference standard used to diagnose asthma

From scoping work, we anticipate that studies designed to derive and validate asthma diagnosis CPMs will use a variety of reference standards. We will evaluate the impact of using different measures as the reference standard. However, studies which do not use an internationally accepted definition for asthma will be excluded.

#### Exclusion criteria

Studies will be excluded if:

1. The predictive value of more than one variable was evaluated but not combined to produce a diagnostic estimate.
2. Publication occurred before 1 January 1990.
3. Variables used in the model are not available in routine clinical practice or not clearly reported.
4. Outcomes for asthma are not separate or data relating to the asthma outcome is not extractable.
5. The CPM was derived to predict future risk of asthma.
6. Over half of the participants included were children less than 5 years old (because of the overlap between asthma and viral-associated wheeze in this age group).
7. The reference standard used is not based on an internationally recognised definition of asthma.
8. Non-original studies such as editorials, expert views, non-research letters.

### Data management and selection process

Records will be retrieved from databases and transferred into a reference manager. Records will be de-duplicated, screened, and managed using Covidence (https://www.covidence.org). Two reviewers (L.D., A.B.) will independently screen titles and abstracts for eligibility. Full-text copies of all potentially relevant records will be obtained. Two reviewers (L.D., A.B. or S.McL.) will independently assess whether each full-text record meets the inclusion criteria. Any discrepancies will be arbitrated by a third reviewer (H.P., S.L., or A.S.).

### Data extraction

Included articles will be categorised into two groups: derivation studies and external model validation (with or without model updating). A standardised data extraction form to collect relevant data items from eligible studies will be developed and piloted in line with Cochrane and CHARMS guidance.^23^ Data extraction will be undertaken by two reviewers (L.D., S.McL.) who will independently extract relevant data from each included study onto the data extraction form. Any disagreements will be resolved by a third reviewer (H.P., S.L., or A.S.).

### Data items

Data from the included studies will be summarised in descriptive tables. Table 1 lists the minimum set of data items to be extracted. If a study reports more than one model, we will extract data separately for each model.

**Table 1:** Minimum data items to be extracted from studies reporting model derivation or model validation

Data item	Model derivation studies	External model validation studies
Study author(s) and year of publication	✓	✓
Country of study and publication language	✓	✓
Source of funding and conflicts of interest	✓	✓
Study design	✓	✓
Study population	✓	✓
Geographical location	✓	✓
Number of participants	✓	✓
Number of outcome events (asthma diagnosis confirmed)	✓	✓
Number and description of candidate predictors	✓	
Definition and method used for measuring the outcome	✓	✓
Number of participants with missing data	✓	✓
Methods for handling missing values (e.g., imputation, complete case analysis)	✓	✓
Methods for internally validating the model (e.g., bootstrapping, split-sample)	✓	
How the model is presented (e.g., full or simplified equation, score chart)	✓	
Measures of model performance (e.g., calibration, discrimination)	✓	✓
Method of external validation (e.g., geographical, temporal)		✓
Study investigators (developers of the original model or not?)		✓

### Critical appraisal and risk of bias in individual studies

The CHARMS checklist will guide risk of bias assessment within individual studies.^23^ The prediction model risk of bias assessment tool (PROBAST) will be used if available.^27^ Quality assessment will be used to evaluate whether study quality makes any impression on the conclusions.

### Data synthesis

Results will be summarised by narrative synthesis and descriptive statistics. Methods for meta-analysis of model validation studies have recently been reported^28^ and will be considered if suitable studies are identified.

### Publication bias

If possible, we will evaluate publication bias using Begg and Egger tests and funnel plots.^29,30^

### Evaluating confidence in cumulative evidence

We will evaluate the overall evidence and strength of recommendations gathered from the review using the Grading of Recommendations Assessment, Development and Evaluation system.^31^

### Protocol registration

A protocol will be registered with the International Prospective Register of Systematic Reviews (PROSPERO). In the event of a protocol amendment, the date, description, and rationale will be reported.

### Data availability

Data sharing is not applicable as this article describes a protocol and no datasets were generated or analysed.

## Electronic supplementary material

Appendix 1
