# Supplementary material for: Clinical prediction models to support the diagnosis of asthma in primary care: a systematic review protocol
Source: NPJ Prim Care Respir Med. 2018 May 18;28:15. doi: 10.1038/s41533-018-0086-6 (PMC5959853; doi:10.1038/s41533-018-0086-6)
Supplement: Supplementary file 1 — Appendix 1 [file 41533_2018_86_MOESM1_ESM.docx]

**Appendix 1: Search strategy for Medline**

| 1 | exp Asthma/ |
| --- | --- |
| 2 | asthma$.mp. |
| 3 | (antiasthma$ or anti-asthma$).mp. |
| 4 | Respiratory Sounds/ |
| 5 | wheez$.mp. |
| 6 | Bronchial Spasm/ |
| 7 | bronchospas$.mp. |
| 8 | (bronch$ adj3 spasm$).mp. |
| 9 | bronchoconstrict$.mp. |
| 10 | exp Bronchoconstriction/ |
| 11 | (bronch$ adj3 constrict$).mp. |
| 12 | Bronchial Hyperreactivity/ |
| 13 | Respiratory Hypersensitivity/ |
| 14 | ((bronchial$ or respiratory or airway$ or lung$) adj3 (hypersensitiv$ or hyperreactiv$ or allerg$ or insufficiency)).mp. |
| 15 | OR/1-14 |
| 16 | (Validat$ OR Predict$.ti. OR Rule$) |
| 17 | (Predict$ AND (Outcome$ OR Risk$ OR Model$)) |
| 18 | ((History OR Variable$ OR Criteria OR Scor$ OR Characteristic$ OR Finding$ OR Factor$) AND (Predict$ OR Model$ OR Decision$ OR Identif$ OR Prognos$)) |
| 19 | (Decision$ AND (Model$ OR Clinical$ OR Logistic Models/)) |
| 20 | (Prognostic AND (History OR Variable$ OR Criteria OR Scor$ OR Characteristic$ OR Finding$ OR Factor$ OR Model$)) |
| 21 | Stratification.mp |
| 22 | ROC curve/ |
| 23 | Discrimination.mp |
| 24 | Discriminate.mp |
| 25 | c-statistic.mp |
| 26 | c statistic.mp |
| 27 | Area under the curve.mp |
| 28 | AUC.mp OR Area Under Curve/ |
| 29 | Calibration.mp OR Calibration/ |
| 30 | Indices.mp |
| 31 | Algorithm.mp OR Algorithms/ |
| 32 | Multivariable |
| 33 | OR/16-32 |
| 34 | Asthma/di |
| 35 | Exp *Diagnosis/ |
| 36 | (diagnos?s or diagnostic).tw. |
| 37 | OR/35-36 |
| 38 | pre-school$.ti. |
| 39 | preschool$.ti. |
| 40 | infant$.ti. |
| 41 | newborn$.ti. |
| 42 | OR/38-41 |
| 43 | 15 and 33 and 37 |
| 44 | 43 not 42 |
| 45 | Animals/ not Humans/ |
| 46 | 44 not 45 |
| 47 | Editorial/ |
| 48 | Letter/ |
| 49 | 47 or 48 |
| 50 | 46 not 49 |
